# Correlation between *BPI* Gene Upstream CpG Island Methylation and mRNA Expression in Piglets

**DOI:** 10.3390/ijms150610989

**Published:** 2014-06-18

**Authors:** Jing Wang, Xuemei Yin, Li Sun, Shouyong Sun, Chen Zi, Guoqiang Zhu, Shenglong Wu, Wenbin Bao

**Affiliations:** 1 Key Laboratory for Animal Genetics, Breeding, Reproduction and Molecular Design of Jiangsu Province, College of Animal Science and Technology, Yangzhou University, Yangzhou 225009, China; E-Mails: jinghostwj@gmail.com (J.W.); 18061151891@163.com (X.Y.); sl19920327@163.com (L.S.); dkxy@yzu.edu.cn (S.S.); zchandy@163.com (C.Z.); slwu@yzu.edu.cn (S.W.); 2 College of Veterinary Medicine, Yangzhou University, Yangzhou 225009, China; E-Mail: yzgqzhu@yzu.edu.cn

**Keywords:** pig, *BPI* gene, CpG island, methylation, BSP (bisulfite sequencing PCR)

## Abstract

Diarrhea and edematous disease are two major causes of mortality in postweaning piglets, and these conditions lead to huge economic losses in the swine industry. *E. coli* F18 is the primary causative agent of these two diseases. Bactericidal/permeability-increasing protein (BPI) plays an important role in the natural defense of the host. The aim of this study was to determine the correlation between *BPI* gene upstream CpG island methylation and mRNA expression. In this study, bisulfite sequencing PCR (BSP) was used to detect the methylation status of the *BPI* gene upstream CpG island and fluorescence quantitative PCR was used to detect *BPI* expression in the duodenum of piglets from birth to weaning age. *BPI* upstream CpG islands were shown to have many putative transcription factor binding sites, 10 CpG sites and every CpG site was methylated. The CpG island methylation level was lowest in 30-day piglets and was significantly lower than levels in 8-day piglets (*p* < 0.05). *BPI* mRNA expression was significantly higher in 30-day piglets than at any other age (*p* < 0.05). Pearson’s correlation analysis showed that the methylation status of the CpG island was negatively correlated with *BPI* mRNA expression. Statistical significances were found in CpG_1, CpG_3, CpG_4, CpG_7 and CpG_10 (*p* < 0.05). The data indicate that *BPI* expression is improved by demethylation of the *BPI* gene upstream CpG island. Furthermore, CpG_1, CpG_3, CpG_4, CpG_7 and CpG_10 may be critical sites in the regulation of *BPI* gene expression.

## 1. Introduction

Bactericidal/permeability-increasing protein (BPI) is an endogenous cationic protein. In addition to killing Gram-negative bacteria and neutralizing endotoxin and lipopolysaccharide (LPS, also known as endotoxin), BPI has several biological functions, such as promoting complement activation and opsonization for increased phagocytosis, inhibiting angiogenesis and the release of inflammatory mediators, as well as protecting against infection by fungi and protozoan pathogens; thus, BPI plays an important role in the natural defense of the host [1]. Schultz, *et al.* [2] have reported that human skin fibroblasts and mucosal cells can increase local BPI protein expression level, thus protecting the local tissue from systemic infection and inflammation. Furthermore, Mao, *et al.* [3] reported that high expression of *BPI* may contribute to host immune defense against Gram-negative bacterial infections in ark shell *Scapharca broughtonii*. In recent years, *BPI* was identified as a candidate gene for disease-resistance breeding in pig [4]. *Escherichia coli* F18 (*E. coli* F18) is a Gram-negative bacteria, with the main component of the cell wall being LPS, which is the main bacterial pathogenic factor [5]. *E. coli* F18 is the primary causative of diarrhea and edematous disease which are two major causes of mortality in postweaning piglets, and these disease lead to huge economic losses in the swine industry [6]. Our preliminary study suggested that *BPI* expression is connected to resistance against *E. coli* F18 [7].

DNA methylation is one of the most common mechanisms of epigenetic regulation, whereby 5-cytosine in guanine and cytosine-rich region (CpG islands) is converted to 5-methylcytosine (5mC) by methyltransferases. DNA methylation occurs mainly in the CpG island-rich promoter region, where it can hinder binding of transcription factors to the promoter, thereby inhibiting gene transcription [8]. In view of the importance of promoter in gene transcription regulation and the close relationship between *BPI* gene expression and *E. coli* F18-resistance, bisulfite sequencing PCR (BSP) was used to detect the methylation status of the *BPI* gene upstream CpG island and fluorescence quantitative PCR was used to detect *BPI* expression in the duodenum of piglets from birth to weaning age. Our objective was to investigate the correlation between *BPI* gene upstream CpG island methylation status and mRNA expression, to provide a theoretical basis for resistance to *E. coli* F18 infection in pig.

## 2. Results and Discussion

### 2.1. Bioinformatic Analysis

**Figure 1:**
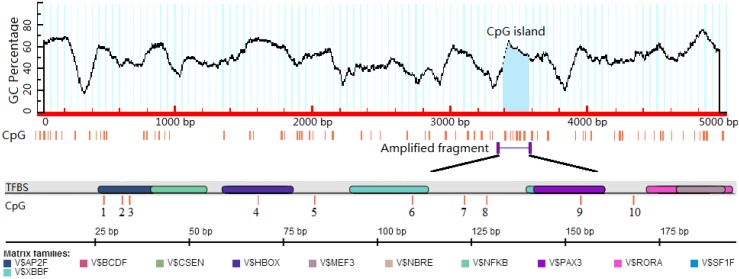
Bioinformatic analysis of the CpG island of the porcine *BPI* gene upstream-5kb region. TFBS: transcription factor binding sites; Matrix Families: similar and/or functionally related TFBS are grouped into so-called matrix families.

The results of MethPrimer analysis showed that the porcine *BPI* gene upstream-5 kb region contains only one CpG island, which contains 10 CpG sites (Figure 1). Therefore, primers were designed for amplification of a fragment containing the whole CpG island. MatInspector was used to identify putative transcription factor binding sites (TFBS) within the CpG island using the following conditions: Core similarity, set to 1.00, Matrix similarity, set to Optimized and greater than 0.90. Twelve putative TFBS were identified (Table 1), six of which contain CpG sites: Ap-2, Gsh-2, CRX-1, RFX-5, RFX-4 and Pax-3.

**Table 1:** Transcription factor binding sites Information. Matrix Families: Similar and/or functionally related TFBS are grouped into so-called matrix families. Matrix similarity: the matrix similarity is calculated as described in the MatInspector papers, a perfect match to the matrix gets a score of 1.00 [9].

Matrix Family		Detailed Matrix Information		Start Position		End Position		Matrix Similarity	
V$AP2F		Transcription factor AP-2, beta		26		40		0.901	
V$NFKB		c-Rel		40		54		0.910	
V$BCDF		Cone-rod homeobox-containing transcription factor/otx-like homeobox gene		60		76		0.976	
V$HBOX		Homeodomain transcription factor Gsh-2		59		77		0.957	
V$XBBF		Regulatory factor X, 5		93		113		0.946	
V$XBBF		Regulatory factor X, 4		140		160		0.922	
V$PAX3		Pax-3 paired domain protein		142		160		0.943	
V$SF1F		SF1 steroidogenic factor 1		174		188		0.996	
V$NBRE		Monomers of the nur subfamily of nuclear receptors (nur77, nurr1, nor-1)		175		189		0.941	
V$RORA		RAR (Retinoic acid receptor)-related orphan receptor alpha1		172		194		0.932	
V$CSEN		Downstream regulatory element-antagonist modulator, Ca^2+^-binding protein of the neuronal calcium sensors family that binds DRE (downstream regulatory element) sites as a tetramer		180		190		0.992	
V$MEF3		MEF3 (Myocyte enhancer factor 3) binding site, present in skeletal muscle-specific transcriptional enhancers		180		192		0.912	

### 2.2. Validation of the CpG Island Fragment Amplification

**Figure 2:**
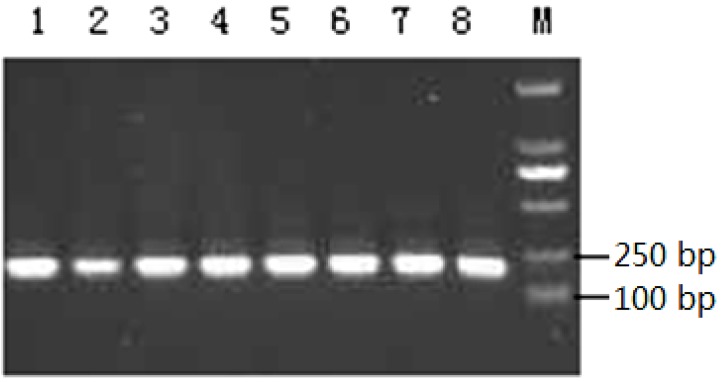
Agarose gel (1%) electrophoresis for *BPI* gene PCR products. Lanes **1**–**8**: *BPI* gene products; **M**: DL2000 molecular weight markers.

The products of BSP primer pair amplification from DNA extracted from the pig duodenum were examined by 1% agarose gel electrophoresis. The size of the amplified fragments corresponded with the expected PCR product sizes (195 bp) and each amplified a single specific product which could be directly cloned and sequenced (Figure 2).

### 2.3. Results and Analysis of Methylation Levels

A total of 237 correct clones of CpG island containing fragments were obtained and confirmed by sequencing; the average number of recombinant clones for each individual was 14.8 (range 12–18). All CpG sites were methylated (Table 2, Figure S1). Overall, the CpG island methylation levels at 8, 18, 30 and 35 days of age were 75.94% ± 26.75%, 61.41% ± 35.14%, 36.04% ± 10.28% and 61.48% ± 23.22%, respectively. A significant difference was found between the levels of methylation at days 8 and 30 (*p* < 0.05). Single CpG site analysis showed significant differences in the methylations levels of CpG_3, CpG_4 and CpG_10 site in the different age groups (*p* < 0.05).

**Table 2:** Average methylation levels of overall and single CpG sites in the porcine *BPI* gene in the different age groups; Figure in the table is mean ± SE; ^a,b^ The means in the same row with different superscripts differ significantly (*p* < 0.05).

Methylation Level (%)	8-Day	18-Day	30-Day	35-Day
Overall	75.94 ± 26.75 ^a^	61.41 ± 35.14 ^a^^,^^b^	36.04 ± 10.28 ^b^	61.48 ± 23.22 ^a^^,^^b^
CpG_1	71.43 ± 28.09	69.73 ± 16.75	42.30 ± 10.44	60.13 ± 34.29
CpG_2	75.55 ± 36.80	62.98 ± 32.53	31.90 ± 14.15	49.58 ± 33.77
CpG_3	85.00 ± 19.15 ^a^	67.93 ± 26.02 ^a^^,^^b^	27.95 ± 18.85 ^b^	58.85 ± 24.54 ^a^^,^^b^
CpG_4	87.58 ± 11.36 ^a^	79.00 ± 16.93 ^a^	41.18 ± 15.92 ^b^	73.70 ± 11.09 ^a^
CpG_5	67.13 ± 35.41	60.00 ± 33.91	25.00 ± 15.23	55.30 ± 25.86
CpG_6	79.23 ± 26.22	57.80 ± 30.84	44.45 ± 18.99	67.85 ± 23.69
CpG_7	65.38 ± 14.05	61.10 ± 27.67	36.40 ± 22.31	63.93 ± 9.54
CpG_8	78.15 ± 25.64	55.33 ± 43.18	47.73 ± 14.08	74.60 ± 25.62
CpG_9	73.98 ± 33.66	57.85 ± 35.36	51.13 ± 5.11	62.03 ± 22.05
CpG_10	68.58 ± 27.33 ^a^	44.58 ± 18.04 ^a^	4.18 ± 8.35 ^b^	50.18 ± 24.72 ^a^

### 2.4. Correlation between the Methylation Levels and mRNA Expression

**Figure 3:**
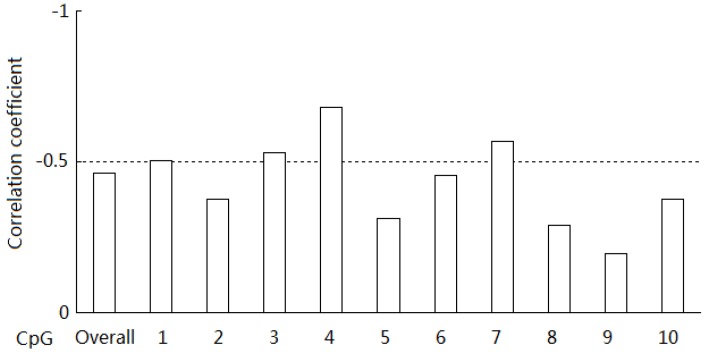
Correlation analysis of methylation levels and mRNA expression at different CpG sites in the porcine *BPI* gene CpG island.

The mRNA expression levels at 8, 18, 30 and 35 days of age were 1.06 ± 0.38, 1.20 ± 0.52, 8.62 ± 6.49 and 1.48 ± 1.48, respectively. Significantly higher levels of *BPI* mRNA expression were detected only in 30-day old piglets compared with the other age groups (*p* < 0.05). Pearson correlation analysis showed that the methylation status of the CpG island was negatively correlated with *BPI* mRNA expression; with significant correlation coefficients for CpG_1, CpG_3, CpG_4 and CpG_7 (*p* < 0.05, Figure 3, Table S1).

### 2.5. Discussion

Promoters are often located in the upstream of genes and methylation in this region is one of the most common epigenetic mechanisms underlying the suppression of gene expression. In general, DNA methylation occurs most commonly in CpG islands, which are located in the promoter or first exon [10]. CpG island is a region with at least 200 bp, and a GC percentage that is greater than 50%, and with an observed-to-expected CpG ratio that is greater than 60% [11]. DNA methylation regulates transcription factor (TF) activity by methylation or demethylation of TFBS located in GC-rich regions, resulting in specific inhibition or activation of gene expression [12,13,14]. The results of MethPrimer analysis showed that the porcine *BPI* gene upstream-5 kb region contains only one CpG island, which contains 12 putative TFBS. These observations indicate that the CpG island is a key region in the regulation of *BPI* gene expression, so this regulation is likely to be multi-factorial and more comprehensive investigations are required for complete elucidation.

Baker-Andresen *et al.* [15] reported that the highly dynamic process of promoter methylation is an important regulatory mechanism of normal developmental processes. Such dynamic methylation processes are particularly evident during embryonic development, and methylation plays a key role in the reprogramming of the genome in early mammalian embryogenesis [16,17,18]. Changes in DNA methylation with aging have also been reported in post-natal animals [19,20,21]. In the present study, we observed methylation of all the CpG sites in the CpG island of the *BPI* gene, and that the pattern of methylation altered dynamically with piglet age. RT-PCR analysis revealed that the methylation status of the CpG island was shown negatively correlated with *BPI* mRNA expression levels, indicating that *BPI* gene expression is suppressed by CpG island methylation. BPI is an endogenous cationic protein which can kill Gram-negative bacteria and neutralize LPS [22]. Combined with the biological activity of BPI, BPI is directly and closely related to resistance to intestinal *E. coli* and other Gram-negative bacteria. When piglets are invaded by *E. coli* strains, the bactericidal effects of BPI are activated. Because it has a cytotoxic effect on Gram-negative bacteria and neutralizes endotoxin, it kills *E. coli* strains before they attach to the small intestine and release intestinal toxins, reducing the probability of diarrhea and edema in infected piglets. Bertschinger *et al.* [23] reported that newborn piglets were more susceptible to infection by *E. coli* F4, while at 30 days (the transition period for weaning), piglets were more susceptible to infection by *E. coli* F18. It can be speculated that at 30 days, *BPI* expression levels are increased in these piglets by CpG island methylation in the intestines leading to *E. coli* F18-resistance.

Barrera *et al.* [24] reported variation in CpG islands in different genome elements. Each CpG island has its own specific function, and only a few CpG sites within each CpG island may be critical for regulation, indicating that methylation of every site is not required for the regulation of gene expression [25]. The BSP cloning sequencing method can be used for analysis of the methylation status of individual CpG sites. In the present study, averages of 15 bacterial colonies were selected for every individual; thus, allowing a high level of precision to be achieved in the analysis of individual CpG sites. Variance analysis showed that significant differences in the methylation status of CpG_3, CpG_4 and CpG_10 among the different age groups (*p* < 0.05). Furthermore, Pearson correlation analysis showed that the methylation status of the CpG island was negatively correlated with *BPI* mRNA expression; correlation coefficients were significant for CpG_1, CpG_ 3, CpG_4 and CpG_7 (*p* < 0.05). These results suggest that all of the CpG sites are involved in the regulation of *BPI* mRNA expression, with CpG_1, CpG_3, CpG_4, CpG_7 and CpG_10 representing critical sites involved in this process.

## 3. Experimental Section

### 3.1. Experimental Animals

The Sutai pig (Duroc (50%) × Meishan (50%) cross) is a new breed of high quality lean-meat type pig bred by the Sutai pig breeding center in Suzhou City (Suzhou, China). It was approved by the National Committee of Livestock and Poultry Species as a new variety in 1999. All pigs (Sutai pigs, 8-, 18-, 30- and 35-day old, *n* = 4 per age group) included in the study were healthy, raised in the same conditions, with similar birth weights, weaning weights, and body sizes. The pigs were purchased from Suzhou Sutai Pig Breeding Center (Suzhou, China) and this experiment was conducted in the Animal Hospital of Yangzhou University according to the regulations of the Administration of Affairs Concerning Experimental Animals (Ministry of Science and Technology, Beijing, China, revised in June 2012). This experiment was approved by the Institution Review Board of the Yangzhou University (permit No. SYXK (Su) 2012-0029).

### 3.2. Bioinformatic Analysis

Analysis and identification of CpG islands and putative TFBS in the *BPI* gene upstream-5 kb region was performed using the online tools MethPrimer and MatInspector [10,26,27].

### 3.3. Methylation Analysis

Genomic DNA was extracted from porcine duodenal tissues by standard phenol/chloroform extraction and subjected to bisulfite conversion using the EpiTect bisulfite kit (Qiagen, Valencia, CA, USA), according to the manufacturer’s instructions. Touchdown PCR was used to amplify the bisulfite-treated DNA (BST-DNA). The primer sequences were: F, 5'-TAAATCGACCCCATCAGCCTC-3' and R, 5'-ATTTAATCCCTAACCTTACTCAATAC-3', the amplified fragment length is 195 bp. The 50 µL reactions included 3 μL DNA template, 3 μL 10× PCR buffer, 2 μL Mg^2+^ (25 mmol/L), 1 μL forward primer (10 μM/L), 1 μL reverse primer (10 μM/L), 1 μL dNTPs (10 mmol/L), 0.8 μL *Taq* polymerase (5 U/μL) and 38.2 μL water. The following reaction conditions were used: 98 °C for 4 min, then 20 cycles of 94 °C for 45 s, 66 °C for 45 s (reduced by 0.5 °C with each cycle) and 72 °C for 1 min; 20 cycles of 94 °C for 45 s, 56 °C for 45 s and 72 °C for 1 min, with a final extension at 72 °C for 10 min. The PCR products were subjected to electrophoresis on agarose gels, excised, purified and inserted into the pMD18-T vector (TaKaRa, Dalian, China). The recombinant clones were used to transform *E. coli* TB1 cells. Positive recombinant clones were selected on LB agar plates containing 100 μg/mL ampicillin, and confirmed by PCR and DNA sequencing (20–30 positive recombinant clones were selected from each individual).

### 3.4. Real-Time PCR Analysis

RNA was isolated from the duodenal tissues of the Sutai pigs using TRIzol reagent (Invitrogen, Carlsbad, CA, USA), according to the manufacturer’s instructions. Single-stranded cDNA was generated using the PrimeScript RT-PCR Kit (TaKaRa) following the manufacturer’s instructions. Real-time quantitative PCR was performed using an ABI Prism 7500 sequence-detection system (Applied Biosystems, Foster City, CA, USA) with SYBR Green PCR Master Mix (TaKaRa), according to the manufacturer’s instructions. The *BPI* fragment was amplified using the primers listed in Table 3 and the *GAPDH* primers were used as internal control; the expression of *BPI* in each sample was normalized to that of *GAPDH*. Triplicate PCR amplifications were performed for each sample.

**Table 3:** RT-PCR (real-time PCR) primers.

Primer	Sequence of Primer	Length (bp)
*BPI* RT-PCR primer	5'-ATATCGAATCTGCGCTCCGA-3'	136
	5'-TTGATGCCAACCATTCTGTCC-3'	
*GAPDH* RT-PCR primer	5'-ACATCATCCCTGCTTCTACTGG-3'	187
	5'-CTCGGACGCCTGCTTCAC-3'	

### 3.5. Data Processing and Analysis

Methylation sequencing results were processed by QUMA software for analysis [28]; the real-time PCR results were processed using the 2^−ΔΔ*C*t^ method (Δ*C*_t_ = mean *BPI* expression − mean *GAPDH* expression) [29]. The average Δ*C*_t_ of the 8-day age group was arbitrarily defined as 1.0 for relative quantification of the expression levels of this gene in the other age groups (ΔΔ*C*_t_ = Δ*C*_t_ of each group − average Δ*C*_t_ of the 8-day age group in each experiment). Statistical analyses were carried out using SPSS 17.0 software (SPSS Inc., Chicago, IL, USA). The LSD (Least Significant Difference) method was used to analyze the significance of differences in methylation level and mRNA expression among the four age groups. Methylation levels and mRNA expression were analyzed by Pearson’s correlation.

## 4. Conclusions

In this study, we identified 12 putative TFBS in the *BPI* gene CpG island, with CpG_1, CpG_3, CpG_4, CpG_7 and CpG_10 implicated as critical sites in the regulation of gene expression. The CpG island methylation status correlated negatively with *BPI* mRNA expression and the pattern of methylation altered dynamically with piglet age. Our data indicate that *BPI* expression is improved in piglets by the demethylation of the *BPI* gene upstream CpG island.

## Supplementary Files

Supplementary File 1 Supplementary Information (PDF, 642 KB)
