# Supplementary material for: Correlation between BPI Gene Upstream CpG Island Methylation and mRNA Expression in Piglets
Source: Int J Mol Sci. 2014 Jun 18;15(6):10989–98. doi: 10.3390/ijms150610989 (PMC4100193; doi:10.3390/ijms150610989)
Supplement: Supplementary File 1 — Supplementary Information (PDF, 642 KB) [file ijms-15-10989-s001.pdf]

Supplementary Information

Figure S1. *BPI* gene methylation levels in piglet age groups. CpG sites are marked with pie charts, black region represent methylation level; [a,b] The means with different superscripts within the same CpG sites differ significantly ($p < 0.05$).

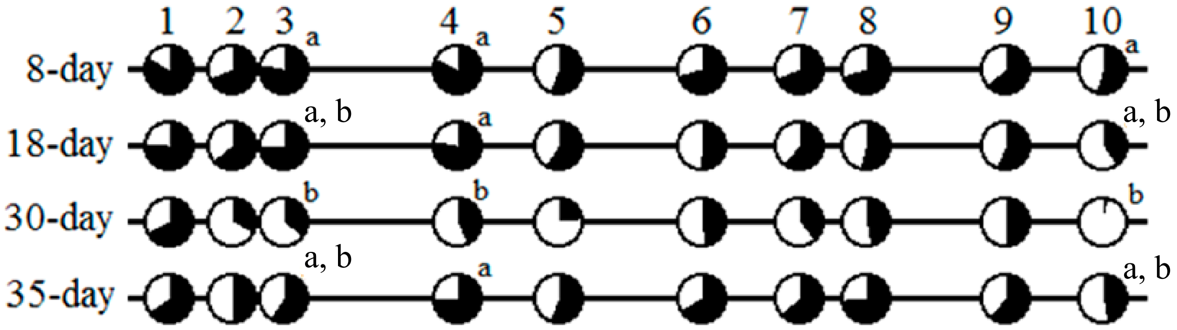

Table S1. Correlation analysis of methylation level and mRNA expression in the porcine *BPI* gene.

CpG Site	Correlation Coefficient	*p*-Value
Overall	−0.463	0.071
CpG_1	−0.503	0.047
CpG_2	−0.376	0.151
CpG_3	−0.529	0.035
CpG_4	−0.680	0.004
CpG_5	−0.312	0.239
CpG_6	−0.455	0.077
CpG_7	−0.567	0.022
CpG_8	−0.291	0.274
CpG_9	−0.194	0.471
CpG_10	−0.377	0.150

© 2014 by the authors; licensee MDPI, Basel, Switzerland. This article is an open access article distributed under the terms and conditions of the Creative Commons Attribution license (http://creativecommons.org/licenses/by/3.0/).
